# The Correlation between Corneal Findings and Disease Severity in Keratoconus per Scheimpflug Corneal Tomography

**DOI:** 10.1155/2020/4130643

**Published:** 2020-02-17

**Authors:** Ahmed E. M. Shehata, James W. Foster, Albert S. Jun, Uri S. Soiberman

**Affiliations:** The Wilmer Eye Institute, Johns Hopkins University School of Medicine, Baltimore, MD, USA

## Abstract

**Purpose:**

This study aims to correlate the clinical signs of keratoconus (KC) which include superficial apical scarring, Fleischer rings, and Vogt striae with best spectacle-corrected visual acuity (BSCVA) and corneal tomography findings. *Patients and methods*. A retrospective observational study. 72 consecutive KC patients seen by the senior author over the course of one year were included in this case series. Eyes with pellucid marginal degeneration, postrefractive ectasia, history of a corneal graft, prior corneal collagen cross-linking, intracorneal ring segments or hydrops were excluded from analysis. Subsequently, the final analysis included only treatment-naïve KC eyes with varying degrees of disease severity.

**Results:**

BSCVA with manifest refraction was 0.5 logMAR higher in eyes with apical scarring (*p* < 0.001). Eyes with apical scarring had worse vision than eyes with Fleischer rings alone (0.43 logMAR higher in the former, *p* < 0.001). Eyes with apical scarring had worse vision than eyes with Fleischer rings alone (0.43 logMAR higher in the former, *K*2 = 64.56 ± 12.89 D versus 49.07 ± 6.61 D, *p* < 0.001). Eyes with apical scarring had worse vision than eyes with Fleischer rings alone (0.43 logMAR higher in the former, *K*2 = 64.56 ± 12.89 D versus 49.07 ± 6.61 D, *p* < 0.001). Eyes with apical scarring had worse vision than eyes with Fleischer rings alone (0.43 logMAR higher in the former, *K*2 = 64.56 ± 12.89 D versus 49.07 ± 6.61 D, *p* < 0.001). Eyes with apical scarring had worse vision than eyes with Fleischer rings alone (0.43 logMAR higher in the former, *p* < 0.001). Eyes with apical scarring had worse vision than eyes with Fleischer rings alone (0.43 logMAR higher in the former, *p* < 0.001). Eyes with apical scarring had worse vision than eyes with Fleischer rings alone (0.43 logMAR higher in the former, *p* < 0.001). Eyes with apical scarring had worse vision than eyes with Fleischer rings alone (0.43 logMAR higher in the former,

**Conclusion:**

The presence of apical scarring and Fleischer rings on biomicroscopy can aid the clinician in making the distinction between severe or long-standing disease (respectively). Apical scarring is a sign of advanced disease and is associated with worse BSCVA and tomography findings. Fleischer rings are markers of intermediate disease and their presence correlates with disease duration.

## 1. Introduction

**Figure 1:**
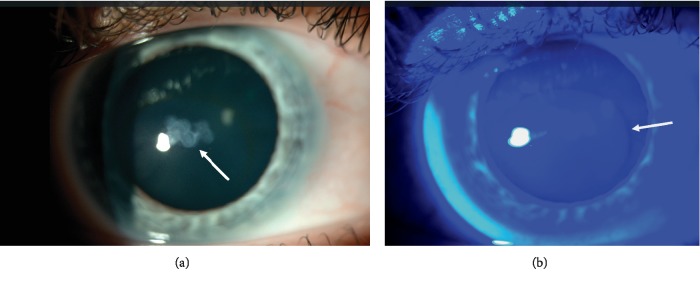
(a) Nodular apical scarring (arrows). (b) Fleischer ring (indicated by the arrow) demonstrated under cobalt blue light.

**Figure 2:**
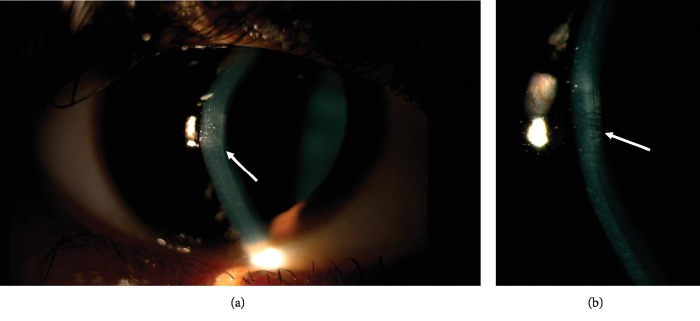
Slit-lamp biomicroscopy showing Vogt striae in a patient with keratoconus as indicated by the arrows.

Keratoconus (KC) is a chronic progressive thinning of the cornea that results in myopia and irregular astigmatism [1, 2]. Common corneal clinical findings in KC include subepithelial apical scarring, Fleischer rings, and Vogt striae (Figures 1 and 2). The collaborative longitudinal evaluation of keratoconus (CLEK) study, the most extensive study to correlate between corneal clinical findings and keratometry measurements, has shown that corneal scarring, Fleischer rings, and Vogt striae are associated with higher corneal curvatures and advanced disease [3]. Scarring has also been associated with worse visual acuity and a higher refractive error [4, 5]. However, corneal curvature measurements were obtained by manual keratometry rather than modern Scheimplug corneal tomography. Since manual keratometry may be less repeatable in KC [6] and does not measure the posterior corneal curvature, this study aims to correlate the presence of specific clinically detected corneal findings in KC, especially superficial apical scarring and Fleischer rings with visual acuity and tomographical indices using contemporary Scheimpflug tomography. By doing so, this study attempts to determine whether observing the abovementioned clinical findings on exam could aid the clinician in determining the stage of keratoconus. Additionally, this study aims to assess the effect of apex-pupil center displacement and visual acuity.

## 2. Materials and Methods

This retrospective study was approved by the Johns Hopkins University School of Medicine institutional review board (IRB). All keratoconus patients seen by the senior author between August 2016 and July 2017 were included in this case series. Eyes with pellucid marginal degeneration, postrefractive ectasia, history of a corneal graft, prior corneal collagen cross-linking, and intracorneal ring segments or hydrops were excluded from analysis. Subsequently, the final analysis included only treatment-naïve keratoconus patients with varying degrees of disease severity.

The diagnosis of KC was based on the presence of findings on biomicroscopy and/or tomography. Biomicroscopic findings consistent with the diagnosis of KC included the appearance of a cone, paracentral stromal thinning, Fleischer rings, scarring, or Vogt striae. Subepithelial (apical) scarring was defined as superficial scarring at the subepithelial-Bowman's layer-anterior stroma level that assumed nodular, reticular, or combined forms. All KC-associated clinical findings were meticulously sought on biomicroscopy during the patients' initial clinic encounter, and their presence or absence was noted. Tomographic findings included inferior (or asymmetrical) steepening with irregular, skewed axes of astigmatism, an inferior-superior index of at least 1.2 Diopters (D), stromal thinning at the area of the cone, and posterior elevation of at least 20 microns [2, 7]. In cases where no biomicroscopic signs were identified, the diagnosis of KC was made solely based on tomographical findings.

Other parameters documented were demographical information, patient reported disease duration from diagnosis, and history of atopic disease (such as asthma, atopic dermatitis, seasonal allergies, or hay fever). All patients underwent corneal tomography using the Oculus Pentacam (Oculus, Germany). In order to facilitate statistical analysis, best spectacle-corrected visual acuity (BSCVA) was converted from Snellen acuity to logMAR with hand motion vision set at 2.3 and counting fingers at 1.85 [8, 9]. BSCVA was used rather than contact lens-corrected visual acuity, as the latter form of correction tends to produce excellent visual results even in severe disease, whereas the former does not, and is therefore more indicative of disease severity. Additionally, the distance of the pupil center from the corneal apex was converted from Cartesian coordinates (*X* and *Y*) to polar coordinates (radius and angle).

### 2.1. Statistical Analysis

All analyses were performed using STATA 14 software (Stata, Inc., College Station, TX). Simple logistic regression models were used to examine the relationship between the presence of each of the biomicroscopic signs of KC and age, ethnicity, disease duration, contact lens wear, and history of atopic disease. Simple linear regression models were also used to compare BSCVA between eyes with or without the abovementioned, specific biomicroscopic signs of KC, to compare Pentacam tomography parameters between eyes with apical scarring and eyes with Fleischer rings but no apical scarring, and to correlate BSCVA to the pupillary distance from the corneal apex. A two-sample *t* test with equal variances was used to compare the mean tomographical findings between eyes with or without specific biomicroscopic signs of KC; a Mann–Whitney test was used for abnormally distributed tomographical parameters. A *p* value of 0.05 or lower was considered statistically significant.

## 3. Results

**Figure 3:**
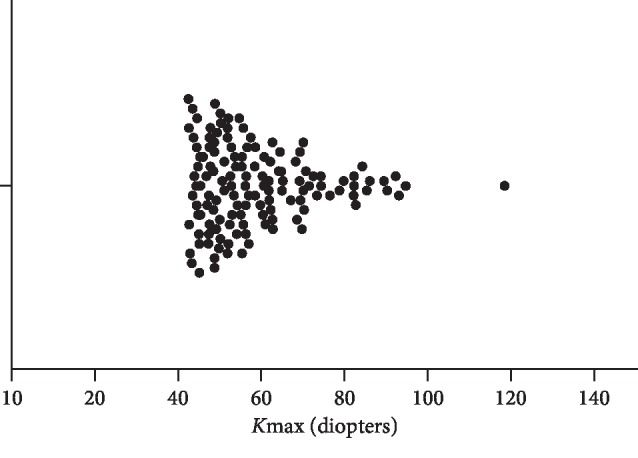
The distribution of maximum simulated keratometry (*K*_max_) values.

134 eyes of 72 patients were included in this study. The mean age of the study subjects was 32.73 ± 13.09 years (mean ± standard deviation), and the range was 12–67 (Table 1 for additional demographical data). As shown in Figure 3, the distribution of disease severity was consistent with that of a tertiary referral center, with most eyes having mild to moderate disease and approximately one-third with severe disease.

A history of atopic disease was present in 27 of the cases included in this series (45.97% of the eyes). Apical scarring was noted in 25 eyes (18.66%), 23 eyes (17.42%) had Vogt striae, and 55 eyes (41.35%) had Fleischer rings. 64 of the eyes in the study had none of the abovementioned clinical signs of KC (47.76%). Apical scarring was the only observed sign in 4 of the eyes, 27 eyes had only Fleischer rings, and Vogt striae was the only observable sign in 8 eyes. Contact lens wear was reported in 43 eyes (32%). Apical scarring was noted to occur concurrently with rigid gas permeable lens use in five eyes, with scleral lens use in five eyes, and with soft contact lens in two eyes.

Apical scarring was an indicator of advanced disease and worse visual acuity: BSCVA with manifest refraction was 0.5 logMAR higher in eyes with apical scarring when compared with eyes without (*n* = 14 versus 78, respectively; 95% CI 0.21−0.78, *p* < 0.001), indicating worse visual acuity. The spherical equivalent was 2.21 D lower in eyes with apical scarring (*n* = 16 versus 81, respectively; 95% CI −3.97 D–−0.45 D, *p* < 0.014), indicating a more myopic refraction. We then assessed whether the presence of Fleischer rings alone predicts a worse visual outcome compared with eyes with apical scarring, with or without other KC clinical signs. Interestingly, almost all Pentacam tomography parameters examined were worse in the latter group indicating that disease severity is worst in eyes with apical scarring (Table 2).

Specifically, BSCVA with manifest refraction was 0.43 logMAR higher in the eyes with apical scarring (*n* = 14 versus 26, respectively; 95% CI 0.11−0.75, *p* < 0.009). Unlike Fleischer rings and apical scarring, the presence of Vogt striae was not associated with worse BSCVA with manifest refraction that was only 0.06 logMAR higher in eyes with Vogt striae (*n* = 14 versus 77; *p*=0.53).

We then assessed whether certain biomicroscopic findings are associated with indicators of advanced KC: higher corneal curvature and lower corneal thickness. Eyes with apical scarring had worse mean anterior and posterior curvature readings. These eyes also had lower mean corneal thickness readings (Table 3). A similar trend was seen in eyes with Fleischer rings compared with eyes without Fleischer rings, as shown in Table 3. Eyes with Vogt striae had higher anterior keratometry indices, as shown in Table 3; however, there were no differences in the steepest posterior curvature, posterior astigmatism, or the thinnest corneal thickness measurements (*p*=NS).

Patient reported optical correction with scleral lenses was more common in eyes with apical scarring (odds ratio: 4.23, *p* = 0.03), which is an indirect indicator of disease severity. This was also true for eyes with Vogt striae, with or without other clinical signs (OR: 3.77, *p* = 0.04). Patient reported scleral lens wear was associated with higher keratometry values compared with the rigid gas permeable (RGP) contact lens group: *K*2 62.2 D ± 11.33 vs. 52.66 D ± 7.8, *p* < 0.02. A similar trend was noted in *K*_max_: 70.3 D ± 13.6 vs. 55.9 ± 5.8, *p* < 0.02.

The presence of Fleischer rings, with or without other biomicroscopic signs, was associated with a worse BSCVA: on average, BSCVA with manifest refraction was 0.23 logMAR higher (95% CI 0.068–0.397) in eyes with Fleischer rings (*n* = 37) compared with eyes without (*n* = 54, *p*=0.006).

We also assessed whether the distance of the pupil center from the corneal apex affects BSCVA. A linear regression model demonstrated a 0.76 logMAR increase per mm distance (*p* < 0.001, *n* = 87), indicating that the farther the corneal apex was from the pupil center, the worse the visual acuity in KC eyes.

The presence of apical scarring, albeit associated with a slightly higher patient age (35.5 ± 11.4 years vs. 32.2 ± 13.4), did not reach a level of statistical significance (*p* = 0.28). This was also true for Vogt striae and Fleischer rings (*p* = 0.41 for both comparisons). We further tested the hypothesis that eyes with longer disease duration were more likely to have characteristic biomicroscopic signs on examination. Neither apical scarring nor Vogt striae were associated with disease duration (*p* = NS), but the odds ratio of Fleischer rings was 1.12 (*p* = 0.002), indicating a 12% increase in risk per year. Additionally, there was no correlation between gender, ethnic background, family history of KC, history of eye rubbing, and presence of either of these clinical signs (*p* = NS for all). However, the odds of having apical scarring were 2.87 times higher in eyes with a history of atopic disease (*p* = 0.03). No correlation was found between Fleischer rings or Vogt striae and atopic disease (*p* = NS).

## 4. Discussion

This study demonstrates that apical scarring in KC is a marker of advanced disease, which is in agreement with the findings of prior studies. Fleischer rings without apical scarring are markers of intermediate disease, at minimum, and so are Vogt striae. Moreover, the presence of Fleischer rings is correlated with disease duration. Together, these findings may provide valuable clinical information on disease duration and severity.

Prior studies have shown that central corneal scarring in keratoconus is associated with advanced disease, higher keratometry, and poor visual acuity [5]. However, scarring was not always meticulously defined as apical or deep scarring [5]. Apical scarring at baseline was associated with older age, but the development of apical scarring over time was not more common in older patients, also consistent with the findings of the present study [5, 10]. The odds ratio of apical scarring has previously been shown to be 4.51 in eyes with manual keratometry readings over 52 D [4]. In the present study, the mean Scheimpflug tomography derived keratometry value for eyes with apical scarring is 61.6 D, suggestive of more advanced disease. This discrepancy can be attributed to method of measurement, as Scheimpflug tomography readings may not correlate well with manual keratometry [11].

In prior studies, Vogt striae and Fleischer rings have been more likely to be present in scarred corneas [4, 10]. However, neither Vogt striae nor Fleischer rings have been shown to be markers of intermediate disease, as shown in the current study. Additional novel findings include the correlation between Vogt striae and Fleischer rings and BSCVA. Specifically, Fleischer rings have been shown to be associated with worse BSCVA.

Additionally, in this dataset of KC patients, the patient reporting optical correction with scleral lenses was associated with higher keratometry values compared with RGP lens wear. As commonly observed in clinical practice, eyes with higher keratometry values (approximately 50–60 D) may not tolerate RGP wear due to apex-contact lens touch. Scleral lenses that generally vault over the central cornea may be better tolerated in this group of patients. However, even with a good fit, eyes with severe KC and very high *K*_max_ values may have unacceptable visual results. These patients remain good candidates for a corneal transplant, either deep anterior lamellar keratoplasty (DALK) or penetrating keratoplasty despite having apical scarring [12]. Posterior stromal scars, which can also be seen in advanced keratoconus, should instigate a thorough examination to rule out Descemet's membrane tears as these generally preclude pneumodissection type DALK [12, 13].

This study demonstrates that optical correction using scleral lenses is more frequent in eyes with apical scarring and Vogt striae, respectively. This finding can be attributed to the fact that patients with more severe disease are more likely to require contact lens for optimum correction; however, the effect of contact lens wear on corneal microstructural abnormalities may possibly contribute to the pathogenesis of superficial corneal scarring [14].

The risk of apical scarring in eyes of atopic patients demonstrated in this study is higher than that previously reported in the literature [4]. One possible explanation for this discrepancy is the difference in the studied populations: the *a priori* probability of severe keratoconus and scarring in our series is higher because more severe cases are often seen in tertiary specialty clinics such as ours. Additionally, the higher likelihood of apical scarring reported in the current study raises the question whether atopic disease is involved in the pathogenesis of this type of scarring seen in KC. Another noteworthy finding is the correlation between disease duration and the presence of Fleischer rings. The deposition of iron in Fleischer rings is a gradual process that occurs over time, and so Fleischer rings are more likely to be visible with higher disease duration [15].

Moreover, progressive displacement of the corneal apex from the pupillary center per corneal tomography has been shown to occur in KC, but this study also associates the degree of displacement with BSCVA [16]. Specifically, for every 1 mm increase in the distance, BSCVA increases in 0.76 logMAR, indicating worse vision [17].

## 5. Conclusions

Overall, this study provides additional information on how clinical findings observed during biomicroscopy can help the clinician to determine the severity, duration, and visual impact of KC. Additionally, this study underscores the importance of corneal tomography in the diagnosis and follow-up of KC patients.

## Figures and Tables

**Table 1:** Demographical data.

Parameter	No. of eyes (%)
Gender	
Male	68 (50.75%)
Females	66 (49.25%)

Ethnicity	
African American	42 (34.43%)
White Caucasian	66 (45.1%)
Asian	6 (4.92%)
Hispanic	8 (6.56%)

Family history of KC	
Positive	18 (17.65%)
Negative	84 (82.35%)

History of atopic disease	
Positive	57 (45.97%)
Negative	67 (54.03%)

History of eye rubbing	
Positive	83 (76.15%)
Negative	26 (23.85%)

Contact lens wear	43 (32.08%)
Rigid gas permeable lens	19 (14.18%)
Scleral lens	12 (8.95)
Soft contact lens	10 (7.46%)
Not specified	2 (1.49%)

**Table 2:** Linear regression analysis for eyes with apical scarring vs. eyes with Fleischer rings but no scarring (*n* = 25 vs. 36).

Parameter	Coefficient	*p* value	95% confidence interval
Front *K*1 (D)	12.9	<0.001	8.396–17.42
Front *K*2 (D)	12.9	<0.001	7.237–18.59
Front *Km* (D)	13.8	<0.001	9.105–18.6
Front astigmatism (D)	2.0	0.009	0.535–3.519
Back *K*1 (D)	−1.7	0.002	−2.664–−0.657
BACK *K*2 (D)	−2.9	<0.001	−4.217–−1.544
Back *Km* (D)	−2.1	<0.001	−3.012–−1.285
Back astigmatism (*D*)	0.4	0.179	−0.171–−0.898
Thinnest pachymetry (*μ*m)	−75.1	<0.001	−113.82–−36.44

*K*1 = flat keratometry, *K*2 = steep keratometry, *Km* = mean keratometry, *D* = diopters.

**Table 3:** Tomographical characteristics of the studied eyes.

	Apical scarring		*p* value	Fleischer rings		*p* value	Vogt striae		*p* value
	+*n* = 25	−*n* = 109		+*n* = 55	−*n* = 78		+*n* = 23	−*n* = 109	
Front *K*1 (D)	59.9 ± 10.5	45.6 ± 5.1	<0.001	51.8 ± 9.9	45.9 ± 6.5	<0.001	51.5 ± 8.5	47.4 ± 7.8	0.024
Front *K*2 (D)	64.6 ± 12.9	49.1 ± 6.6	<0.001	56.2 ± 11.5	48.9 ± 7.8	<0.001	56.2 ± 10.3	50.7 ± 9.2	0.012
Front *Km* (D)	62.7 ± 11.1	47.1 ± 5.4	<0.001	53.9 ± 10.4	47.4 ± 7.0	<0.001	53.7 ± 9.2	49.0 ± 8.3	0.014
Front astigmatism (D)	5.8 ± 3.2	3.2 ± 2.5	<0.001	4.4 ± 2.5	3.0 ± 2.5	0.002	4.7 ± 3.0	3.3 ± 2.4	0.02
Back *K*1 (D)	−8.4 ± 2.34	−6.5 ± 0.9	<0.001	−7.3 ± 1.7	−6.6 ± 1.2	0.01	−7.7 ± 1.6	−6.7 ± 1.5	0.004
Back *K*2 (D)	−10.0 ± 2.2	−7.1 ± 1.9	<0.001	−8.2 ± 3.0	−7.2 ± 1.4	0.02	−7.8 ± 4.1	−7.5 ± 1.6	0.627
Back *Km* (D)	−9.2 ± 2.0	−6.9 ± 1.0	<0.001	−7.9 ± 1.7	−6.9 ± 1.3	<0.001	−8.1 ± 1.7	−7.1 ± 1.5	0.006
Back astigmatism (D)	1.2 ± 1.3	0.7 ± 0.5	<0.001	1.0 ± 0.9	0.6 ± 0.4	<0.001	0.9 ± 0.4	0.7 ± 0.6	0.266
Thinnest pachymetry (*μ*m)	368.1 ± 65.1	469.2 ± 66.4	<0.001	417.2 ± 84.3	473.9 ± 62.2	<0.001	434.2 ± 66.8	455.2 ± 78.2	0.232

*K*1 = flat keratometry, *K*2 = steep keratometry, *Km* = mean keratometry, *D* = diopters.

## Data Availability

The data used to support the findings of this study are available from the corresponding author upon request.
